# Administration of Glucosylceramide Ameliorated the Memory Impairment in Aged Mice

**DOI:** 10.1155/2013/824120

**Published:** 2013-04-04

**Authors:** Yeonju Lee, Sergiy Oliynyk, Jae-Chul Jung, Jeong Jun Han, Seikwan Oh

**Affiliations:** ^1^Department of Neuroscience and TIDRC, School of Medicine, Ewha Womans University, Seoul 158-710, Republic of Korea; ^2^Institute of Life Science Research, Rexgene Biotech, Ochang, Chungbuk 363-883, Republic of Korea; ^3^Advanced Institutes of Convergence Technology, Doosan Corporation, Glonet BG Biotech Division, Suwon, Gyeonggi-Do 443-270, Republic of Korea

## Abstract

The function and the role of glucosylceramide have not been well studied in the central nervous system. This study was aimed to investigate the possible roles of glucosylceramide in memory function in aged mice. Glucosylceramide (50 mg/kg, p.o.) showed memory enhancing activity after 3-month treatment in the aged mice (C56BL/6, 18–20 months old) through Y-maze, novel objective test, and Morris water maze test. Long-term treatment of glucosylceramide decreased the expression of iNOS and COX-2 in the brain of aged mice. The LPS-induced mRNA level of iNOS, COX-2, IL-1**β**, and TNF-**α** was reduced by the acute treatment with glucosylceramide in adult mice. These results suggest that glucosylceramide plays an important role in anti-inflammatory and memory enhancement, and it could be a potential new therapeutic agent for the treatment of neurodegenerative diseases such as Alzheimer's disease.

## 1. Introduction

Age-related deterioration of brain function produces a variety of behavioral deficits. Age-related impairments in cognitive function include memory and spatial ability [1]. During normal aging the brain undergoes many changes resulting in a detectable cognitive decline that is associated with limited neuronal loss, glial proliferation in the cortex [2, 3]. On the molecular level, the mechanisms about aging of the brain are not yet understood. But aging is a biological process characterized by time-dependent functional declines that are influenced by changes in inflammatory reactions. An organism's proinflammatory status may underlie the aging process and age-related diseases. Chronic inflammation in aging is related to age-associated disorders (e.g., Alzheimer disease, etc.). The pathophysiological events involved in the neuronal dysfunction and degeneration in aging are considered to be the increased inflammation [4]. Neuroinflammation, which develops with age, is closely related to neuronal degeneration and cognitive impairment [5]. The neuroinflammation includes that microglial and astrocytic responses release reactive oxygen species (ROS), nitric oxide, excitatory amino acids, and cytokines, which may result in neurodegeneration [5, 6]. A link between systemic inflammation and dementia was first hypothesized after discovery of upregulated inflammatory processes localized to Alzheimer's disease (AD) pathology in postmortem brain specimens [7]. In cross-sectional analysis of clinical populations, a reasonably consistent finding has been an association between dementia and higher levels of IL-1*β*, IL-6, C-reactive protein, and TNF-*α* [8]. Therefore certain compound that has the anti-inflammatory properties can be a good candidate for healthy aging therapy [9]. The aged mice models have been used to screen for potential treatments for cognitive dysfunction.

In regard to brain functions, the major findings have emphasized the significance of sphingolipids as bioactive molecules that control diverse cellular processes such as proliferation, differentiation, growth, migration, and apoptosis [10]. Sphingolipid metabolites especially, such as ceramide and sphingosine 1-phosphate (S1P), have received much attention as key regulators of cell death and survival [11]. In sphingolipid pathway, sphingosine is phosphorylated to form S1P by sphingosine kinases. Because the phosphorylation of sphingosine is the only pathway for the formation of S1P, cellular S1P is highly dependent on the availability of sphingosine generated by ceramidases. Ceramide mediates a wide array of the stress signals leading to growth arrest or cell death, whereas S1P exerts prosurvival capabilities by antagonizing ceramide effects [12]. The major sphingoid base of mammalian cells is sphingosine and dihydrosphingosine. 

**Figure 1:**
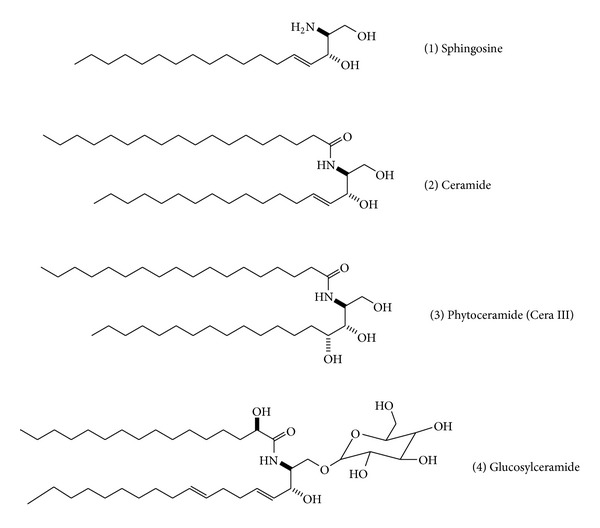
Structures of sphingosine, phytoceramide, and glucosylceramide.

Our previous studies showed that phytoceramide ameliorated the scopolamine-induced memory impairment and showed neuroprotection [13]. Structurally, sphingosine is primary amine containing primary and secondary alcohol groups while ceramide has amide group with alcohols. Ceramide is at the center of sphingolipid metabolism and has been recognized as a critical second messenger. In addition, glucosylceramide is composed of glucose group at primary alcohol position on the ceramide backbone (Figure 1), and glucosylceramide is a major sphingolipid in plants such as soybean, corn, rice, and wheat [14]. Glucosylceramide has recently attracted interest since the beneficial effects of glucosylceramide in improving the skin barrier function by dietary administration as well as topical application [15, 16]. Recently, it is an interesting report that oral administration of glucosylceramide strongly suppressed mRNA expression of the proinflammatory cytokines IL-1*β* and IL-6 in mice [17].

A direct linkage to the sphingolipid metabolism with the neuronal function has not been established well, and it is not understood how such lipid metabolites lead to neuronal dysfunction or survival. Therefore, the current study focused on the effects of the glucosylceramide on the neuronal functions and made a search for the evidence on the relationship between the glucosylceramide and neuronal functions in memory impairment after oral administration. To achieve the objective of the study, the memory performance was examined by using several behavioral tasks in aged mice which were impaired in memory.

## 2. Materials and Methods

### 2.1. Animals and Drug Treatment

The male C57BL/6 mice (28–30 g) were purchased from the Orient Lab Animal (Seoul, Republic of Korea). Mice allowing access to water and food ad libitum were grouped 5-6 per cage and maintained at an ambient temperature of 23°C and a 12 h diurnal light cycle (light on 07:00–19:00). Mice were raised and housed at the laboratory in transparent polycarbonate cages until 18–20 months old. Mice were given experimental diet pellets which contains glucosylceramide for 3 months. All behavioral experiments were carried out in a room adjacent to that in which the mice were housed under the same conditions of temperature and light cycle. All the experiments were carried out using male C57BL/6 mice according to the guidelines of the Animal Care and Use Guidelines of School of Medicine, Ewha Womans University, Republic of Korea.

### 2.2. Glucosylceramide

The plant ceramide (glucosylceramide) from soybean origin was kindly provided by Doosan Co., Glonet Biotech Division (Suwon, Republic of Korea) [17]. The applied glucosylceramide formula contains 61.3% glucosylceramide, 11.5% steryl glycoside, 7.4% phosphatidylcholine, 6.2% phosphatidylinositol, 3.5% triglyceride, 3.5% lysophosphatidylcholine, 2.4% free fatty acid, and so forth. The glucosylceramide was comprised primarily of ceramide with 4,8-sphinganine (d18:2) and alpha-hydroxypalmitic acid (h16:0).

Glucosylceramide was readily characterized through physicochemical instruments after purification by flash column chromatography. Flash column chromatography was performed with Merck silica gel 60 (230–400 mesh) for purification. ^1^H NMR and ^13^C NMR spectra were recorded on Bruker DPX 400 at 400 MHz and 100 MHz, respectively. Proton chemical shifts are reported in ppm relative to internal tetramethylsilane (TMS) or with the solvent reference relative to TMS employed as the internal standard (CDCl_3_). Data are reported as follows: chemical shift {multiplicity [singlet (s), doublet (d), triplet (t), quartet (q), and multiplet (m)], coupling constants [Hz], integration}. Carbon chemical shifts are reported in ppm relative to TMS with the respective solvent resonance as the internal standard (CDCl_3_). Infrared (IR) spectra were recorded on a JASCO FT/IR-430 spectrometer. Data are reported in wave numbers (cm^−1^). Melting Points were determined on a BIBBY Stuart Scientific Melting Point Apprataus SMP3. Mass spectra were recorded with a Waters Micromass ZQ LC-Mass system and high resolution mass spectra (HRMS) were measured with a Bruker BioApex FTMS system by direct injection using an electrospray interface (ESI).

Glucosylceramide: mp: 195-196°C; IR (neat, NaCl) 3353 (O–H), 2920 (C–H), 2851, 1726 (C=O), 1620 (C=C), 1530, 1466, 1406, 1378, 1264, 1182, 1168, 1040 (C–O), 721 cm^−1^; ^1^H NMR (400 MHz, CDCl_3_) *δ* 5.60–5.52 (m, 2H), 5.42–5.28 (m, 2H), 5.01–4.87 (m, 1H), 4.55–4.48 (m, 1H), 4.13–4.09 (m, 1H), 4.04–3.99 (m, 1H), 3.85–3.78 (m, 2H), 3.54–3.28 (m, 6H), 2.50 (brs, 6H), 2.05–1.90 (m, 6H), 1.54–1.45 (m, 2H), 1.33–1.19 (m, 38H), 0.88–0.81 (m, 6H); ^13^C NMR (100 MHz, CDCl_3_) *δ* 173.75, 132.31, 130.23, 129.73, 127.76, 76.89, 76.53, 73.11, 71.59, 70.01, 33.91, 33.22, 32.54, 31.99, 31.29, 30.89, 29.03, 28.71, 28.58, 26.59, 25.21, 24.43, 22.09, 21.96, 14.11; HRMS calcd. for C_40_H_76_NO_9_: 714.5520 [M+H]^+^, found: 714.5531.

### 2.3. Behavioral Test for Learning and Memory

#### 2.3.1. Y-Maze Test

Spontaneous spatial recognition in the Y-maze was used as a hippocampus-dependent test. The Y-maze is a three-arm horizontal maze (40 cm long and 3 cm wide with 12 cm high walls) in which the arms were symmetrically disposed at 120° angles from each other. The maze floor and walls were constructed from dark opaque polyvinyl plastic. Mice were initially placed within one arm, and the sequence (i.e., ABCAB, etc.) and number of arm entries were recorded manually for each mouse over 8-minute period. An actual alternation was defined as entries into all three arms on consecutive choices (i.e., ABC, CAB, or BCA but not BAB). Maze arms were thoroughly cleaned between tasks to remove residual odors. One hour after the last administration of glucosylceramide or saline alone, memory impairment was tested. Mice were gently placed in the maze. The percentage of alternations was defined according to the following equation: %alternation = [(number of alternations)/(total arm entries − 2)] × 100. The number of arm entries served as an indicator of locomotor activity.

#### 2.3.2. Novel Object Recognition

This test was used to measure for objective recognition. The arena was a cage bottom (30 × 40 × 20 cm) with black walls. The objects were the same size but differing in shape, color, and surface texture. On day 1, each mouse was habituated individually to the test box for 8 min sessions in which the animals were allowed to freely explore the open field box. After 6 h, two identical objects were placed in each corner and the mouse was allowed 8 min to explore the objects. Pairs of each object were used for an equal number of times. On day 2 one object was replaced with a novel object in a counter-balanced fashion with respect to object, side, and genotype. The mouse was allowed 8 min to explore the familiar and novel object while it was video-recorded. Exploration was defined as sniffing the object or having one or both forepaws touching the object. From the absolute exploration duration (*T*) an exploration index was calculated: [(*T*_Novel_ − *T*_Familiar_)/(*T*_Novel_ + *T*_Familiar_)] × 100. This measure is considered as an index of recognition memory and takes into account individual differences in the total amount of object exploration time.

#### 2.3.3. Morris Water Maze Test

Young and aged mice were tested in a standardized assessment of spatial cognition prior to behavioral studies with Morris water maze protocol as described in detail elsewhere [18]. The Morris water maze is a circular pool (90 cm in diameter and 45 cm in height) with a featureless inner surface. The pool was filled to a depth of 30 cm with water containing 3000 mL of milk (20°C). The tank was placed in a dimly lit, soundproof test room with various visual cues. The pool was conceptually divided into quadrants. A white platform (6 cm in diameter and 29 cm high) was then placed in one of the pool quadrants and submerged 1 cm below the water surface so that it was invisible at water level. The first experimental day was dedicated to swimming training for 60 sec in the absence of the platform. During the four subsequent days the mice were given four trials per day with the platform in place. When a mouse located the platform, it was permitted to remain on it for 10 sec. If the mouse did not locate the platform within 60 sec, it was placed on the platform for 10 sec. The animal was taken to its home cage and was allowed to dry up under an infrared lamp after each trial. The time interval between each trial was 30 sec. During each trial, the time taken to find the hidden platform (latency) was recorded using a video camera-based Ethovision System (Nodulus, Wageningen, The Netherlands). For each training trial, mice were placed in the water facing the pool wall at one of the pool quadrants in a different order each day. One day after the last training trial sessions, mice were subjected to a probe trial session in which the platform was removed from the pool, allowing the mice to swim for 60 sec to search for it. A record was kept of the swimming time in the pool quadrant where the platform had previously been placed. Glucosylceramide (50 mg/kg, p.o.) was given 1 h before the first trial session at every consecutive day. Control group received saline only.

### 2.4. Immunoblot Analysis

The brain tissue was homogenized with homogenization buffer (0.25 M sucrose, 10 mM Tris-cl pH7.4, 0.5 mM EDTA, 1 mM PMSF, and 1 mM Na_3_VO_4_) and centrifuged at 13500 rpm for 15 min twice at 4°C. Samples were assayed for protein concentration using protein assay kit (Pierce Chemical, Rockford, IL, USA). Proteins were separated by SDS-polyacrylamide gel electrophoresis and transferred to a polyvinylidene difluoride membrane. The membrane was blocked with 5% nonfat dry milk in Tris-buffered saline/Tween 20 solution. The blots were incubated with the iNOS and COX-2 (Millipore Technology Inc., Danvers, MA, USA). GAPDH (Santa Cruz Biotechnology, Inc., Santa Cruz, CA, USA) was performed as an internal control. After washing with Tris-buffered saline/Tween 20, horseradish peroxidase-conjugated secondary antibodies (Cell Signaling Technology Inc.) were applied, and the blots were developed using the enhanced chemiluminescence detection kit (GE Healthcare, Chalfont St. Giles, Buckinghamshire, UK).

### 2.5. Polymerase Chain Reaction

Mice were stimulated with LPS in the absence or presence of glucoceramide for 18 h. Total RNA was isolated from hippocampus of mice using TRIzol (Invitrogen) according to the manufacturer's instructions. For cDNA synthesis, 2 ug of total RNA was reverse-transcribed using the SuperScript First-Strand Synthesis System (Invitrogen). cDNA was amplified by polymerase chain reaction (PCR) using primers for iNOS (F: GTGTTCCACCAGGAGATGTTG, R: CTCCTGC CCACTGAGTTCGTC), COX-2 (F: AAGACTTGCCAGGCTGAACT, R: CTTCTGC AGTCCAGGTTCAA), IL-1*β* (F: AGCAACGACAAAATACCTGT, R: CAGTCCAGC CCATACTTTAG) and TNF-*α* (F: TGTCTCAGCCTCTTCTCATT, R: GTATGAGATA GCAAATCGGC); PCR products were separated by 1% agarose gel electrophoresis and visualized by ethidium bromide staining.

### 2.6. Statistical Analysis

All values were expressed as mean ± standard error (SE). The results were subjected to an analysis of the variance (one-way ANOVA) using the Newman-Keuls multiple comparison test. Differences with *P < 0.05 were considered as statistically significant to analyze the difference.

## 3. Results

### 3.1. Amelioration of the Memory Deficits in Aged Mice through Y-Maze Test

**Figure 2:**
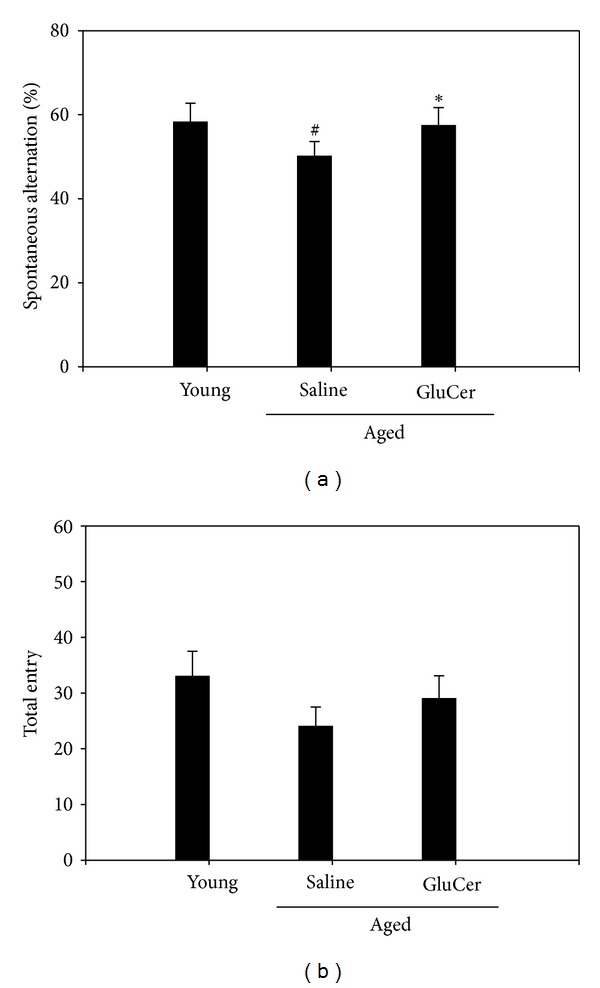
Effects of glucosylceramide on the memory deficit aged mice through Y-maze test. One hour after the administration of glucosylceramide (50 mg/kg) or saline alone, memory impairment was tested. Mice were gently placed in the maze. Mice were initially placed within one arm, and the sequence (i.e., ABCAB, etc.) and number of arm entries were recorded manually for each mouse over 8-minute period. The percentage of alternations was defined according to the following equation: %alternation = [(number of alternations)/(total arm entries − 2)] × 100. The number of arm entries served as an indicator of locomotor activity. Results are expressed as means ± S.E.M. *n* = 8 in each group. ^#^ *P* < 0.05 in comparison with young, *P < 0.05 in comparison with saline group (*n* = 8 mice in each group).

To determine whether glucosylceramide modulate the memory function, aged mice (C57BL/6 male, 18–20 months old) were tested in the Y-maze test. Mice were administrated for 3 months with glucosylceramide (50 mg/kg) which mixed in the chow. Aged mice showed the impairment in working memory function since aged mice spent less spontaneous alteration than young mice in Y-maze test. Glucosylceramide enhanced spatial memory since glucosylceramide-treated mice spent higher spontaneous alteration in the novel arm than that of saline group [*F* (2,21) = 8.388, *P* = 0.0021, one-way ANOVA]. Glucosylceramide resists the working memory retention. There was significant effect on the percentage of alternation (Figure 2).

### 3.2. Amelioration of the Memory Deficits in Aged Mice through Novel Object Task

**Figure 3:**
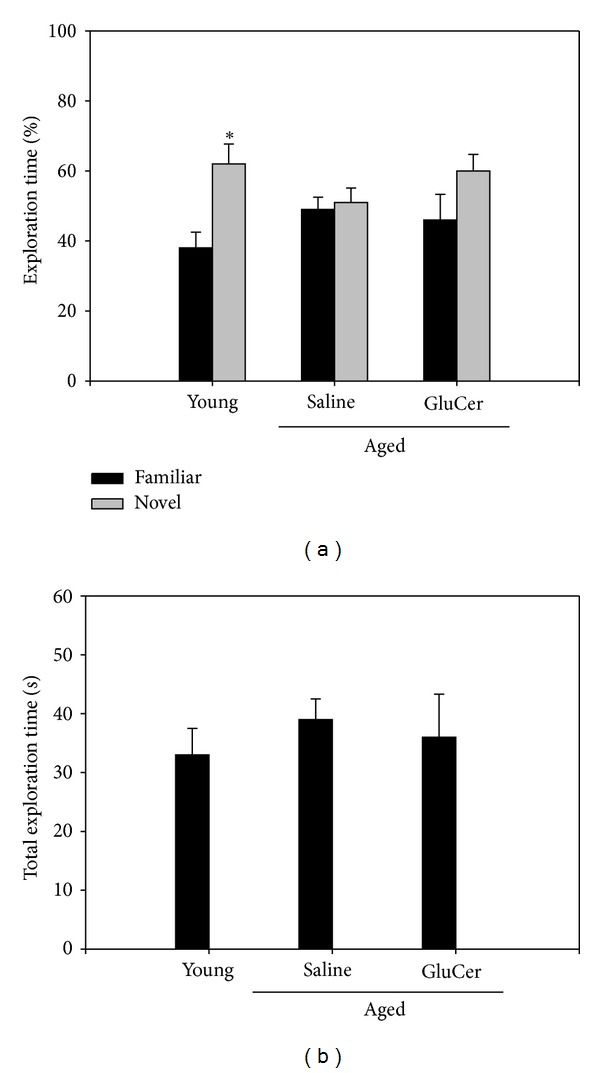
Performance of a novel object recognition task in young and aged mice. (a) The training [(*T*_Novel_ − *T*_Familiar_)/(*T*_Novel_ + *T*_Familiar_)] × 100 session; (b) the test session conducted 24 h after the training session. During the testing session of the novel object recognition memory task, mice treated with saline or glucosylceramide. Mice were administrated with the glucosylceramide (50 mg/kg) for 3 months. Results are expressed as means ± S.E.M. (*n* = 8 in each group).

The amount of time spent with the novel object compared with the total time spent exploring both object represents an index of long-term memory. Mice were administrated with glucosylceramide for 3 months (50 mg/kg). Treatment of glucosylceramide enhances memory function on the performance of a novel object recognition task (Figure 3). As expected, in the training session (Figure 3(a)), the aged mice groups spent similar times investigating each of the identical objects and there were no differences in total exploration times between the test groups. During training, animals showed no preference for one object over another and there was no difference between aged mice in the exploration time, suggesting that the experimental groups were on average equally motivated to explore objects (Figure 3(b)). However, when presented with a novel object, glucosylceramide-treated mice showed a preference for the new one after 24 h of retention [*F* (2,21) = 11.41, *P* = 0.004, one-way ANOVA]. 

### 3.3. Amelioration of the Memory Deficits in Aged Mice through Morris Water Maze Test

**Figure 4:**
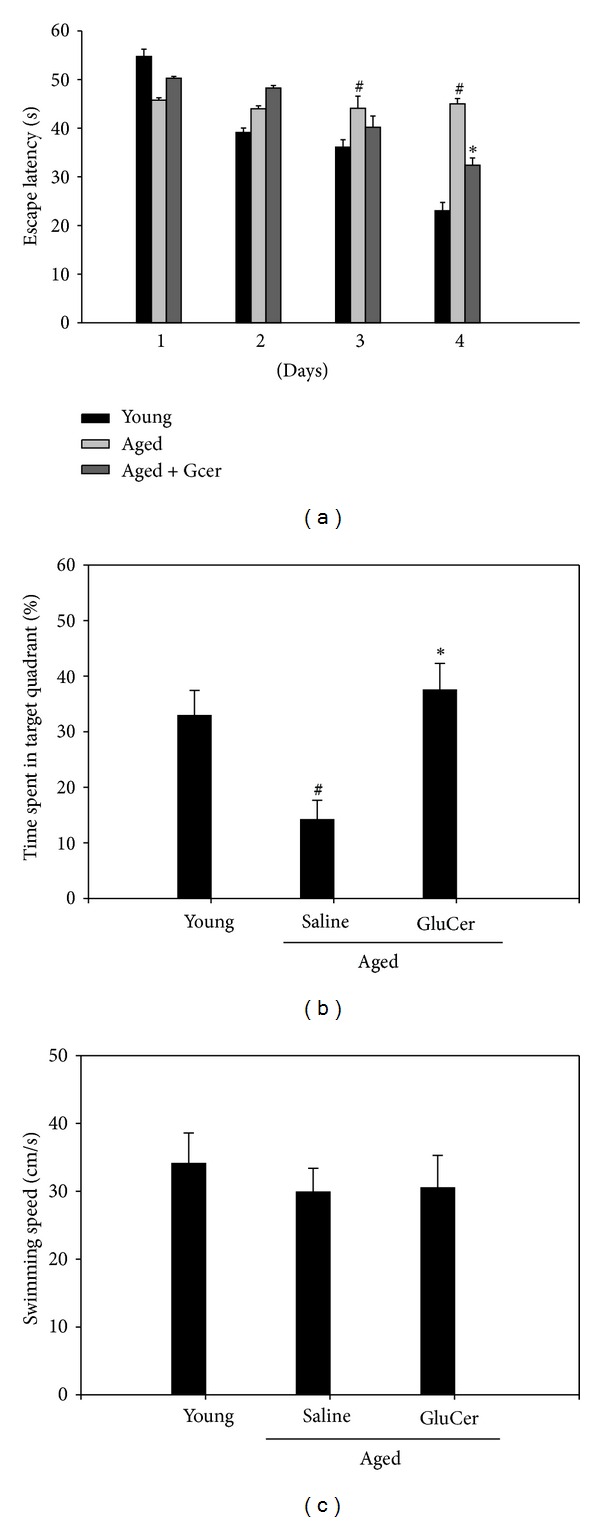
Effect of glucosylceramide on the memory deficit in aged mice through Morris water maze task. Glucosylceramide (50 mg/kg) or vehicle (saline) was orally administered to mice for 3 months before training trial session. The training trial and the probe trial sessions were performed as described in Section 2. Results are expressed as means ± S.E.M. ^#^ *P* < 0.05 in comparison with young group, *P < 0.05 in comparison with saline group (*n* = 8 in each group).

The effects of glucosylceramide on learning and spatial memory were evaluated by the Morris water maze test. Mice were administrated with glucosylceramide (50 mg/kg) for 3 months. The aged group exhibited longer escape latencies throughout the training days than that of the young group. Glucosylceramide group significantly shortened the escape latencies which was prolonged by aged group (*P* < 0.05). On the day following the final day of training trial sessions, swimming times within the platform quadrant for the glucosylceramide group were significantly lower than those of the vehicle-treated young group (*P* < 0.05) (Figure 4). Moreover, the shortened swimming time within the platform quadrant in aged group was significantly increased in glucosylceramide group [*F* (2,21) = 17.69, *P* = 0.0001, one-way ANOVA]. However, no significant differences in swimming speeds were observed between the groups. In this experiment, aged mice increased the escape latency time at the training sessions; however, glucosylceramide treatment shortened this escape latency time on day 4. At the probe trial session, glucosylceramide treatment increased the swimming time within the target quadrant. The decrease in escape latency from day to day in the first trial represents long-term memory or reference memory, while that from first trial of sessions to second trial of sessions represents short-term memory or working memory [19]. The time in the quadrant with the platform reflects changes in spatial memory [20]. These results suggest that glucosylceramide improves the long-term memory in amnesic aged mice. 

### 3.4. Decreasing the iNOS and COX-2 Protein Expression by Long-Term Glucosylceramide Administration in Aged Mice

**Figure 5:**
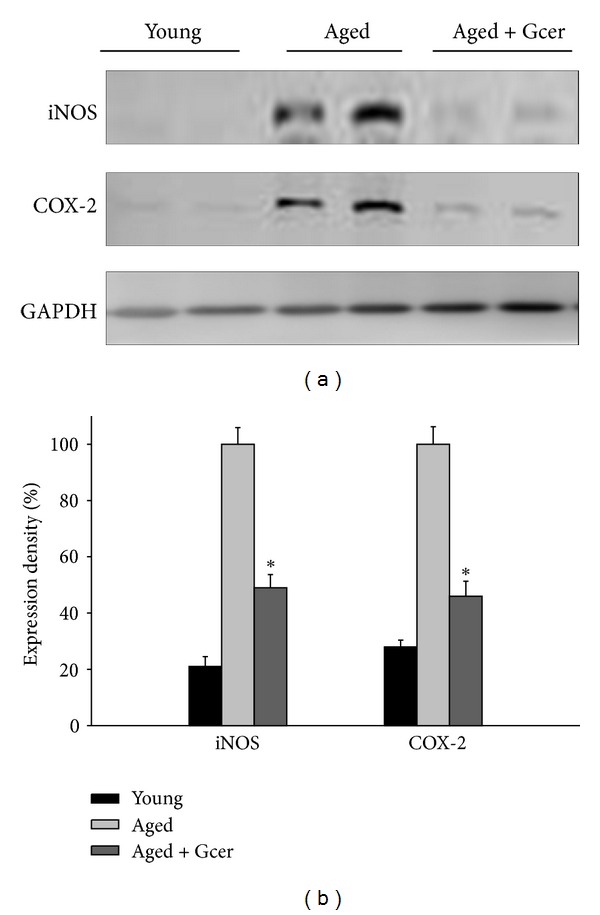
Effect of glucosylceramide on the inflammation-related signals in the hippocampus of mice. Mice were administered with glucosylceramide (50 mg/kg) for 3 months. The expression of iNOS and COX-2 in hippocampus of brain was measured by immunoblot analysis. Glucosylceramide treatment decreased the expression of iNOS and COX-2 in aged mice. GAPDH was used as an internal control. All values are expressed as mean ± S.E.M. from two independent experiments (*n* = 4). *P < 0.05 indicates significant difference between the aged group and glucosylceramide-treated group.

Glucosylceramide exerted an anti-inflammatory effect on age-related responses accompanied by the expression of iNOS and COX-2. Aged mice were administrated with glucosylceramide for 3 months. The protein expression levels of the iNOS and COX-2 in hippocampus of brain were reduced by administration of glucosylceramide (Figure 5). These results indicated that glucosylceramide had an anti-inflammatory effect on the expression of age-related proinflammatory signals in aged mice.

### 3.5. Decreasing the iNOS, COX-2, and Proinflammatory Cytokines mRNA Expression by Glucosylceramide Treatment in LPS-Induced Mice

**Figure 6:**
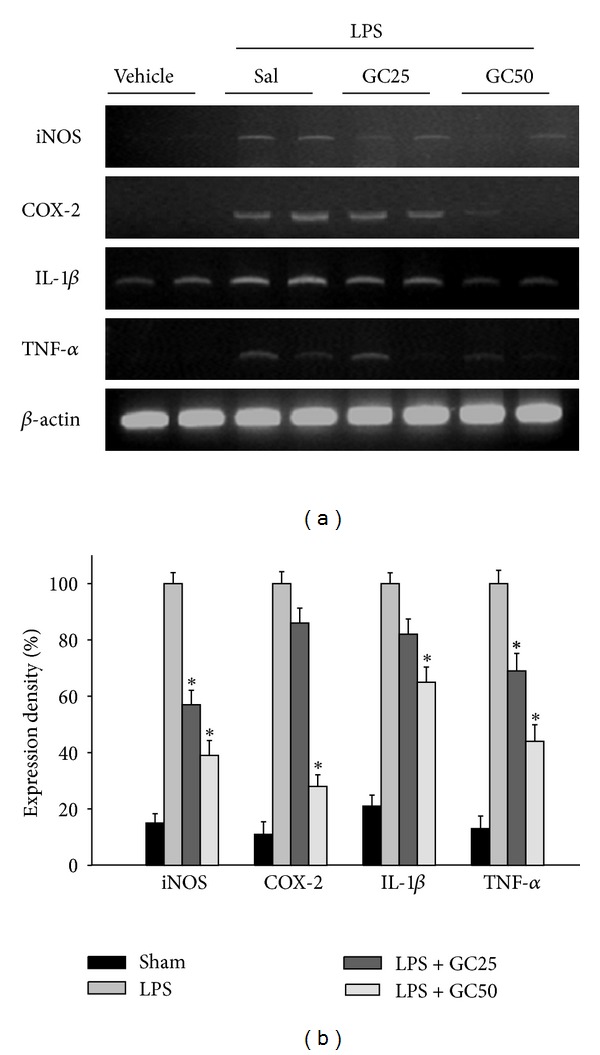
Effect of glucosylceramide on pro-inflammation-related signals in hippocampus of mice. Adult mice were treated with 1 mg/kg LPS (i.p.) to induce inflammation and glucosylceramide (GC 25, 50 mg/kg, p.o.) was pretreated 1 hr before LPS injection. The expression of mRNA of iNOS, COX-2, IL-1*β*, and TNF-*α* was measured at 18 h after LPS injection by immunoblot analysis. *β*-actin was used as an internal control. All values are expressed as mean ± S.E.M. from two independent experiments (*n* = 4). *P < 0.05 indicates significant difference between the LPS group and glucosylceramide-treated group in aged mice.

Glucosylceramide exerted an anti-inflammatory effect on LPS-induced responses accompanied by the expression of proinflammatory cytokines in adult mice. Hippocampus of mice was collected 18 h after treatment of glucoceramide and LPS. The mRNA expression levels of the iNOS, COX-2, IL-1*β*, and TNF-*α* were reduced by treatment with glucosylceramide (Figure 6). These results indicated that glucosylceramide has an anti-inflammatory effect on the expression of LPS-induced proinflammatory cytokines in the brain of mice.

## 4. Discussion

Aging is an important risk factor for Alzheimer's disease (AD) [21, 22]. But the factors that cause the relatively benign process of normal brain aging to the pathological cascade that leads to AD are unknown. Age-related deterioration of brain function produces a variety of behavioral deficits like age-related impairments in cognitive functions, including memory and spatial ability. Recently, neuroinflammatory processes have been identified as key early events strongly implicated in cognitive dysfunction linked to age [23]. The inflammatory cytokine was initially found to be increased in blood of healthy aged humans [24] and mice [25]. Support of this theory comes from studies reporting a progressive, age-associated increase in activated microglia. Alterations in microglial function have been linked to the development of neurodegenerative diseases [26]. Aging changes have been hypothesized to drive the pathogenic progression through a diminution of neuroprotective functions, direct increases in neurotoxicity, and dysregulated responses to signals [27–29]. Age-related alterations previously are characterized in some changes in microglia including changes in cytokine production [30–34], increased expression of activation markers [35–38]. This is in accordance with results obtained in our experiment (unpublished data) and others by showing that inflammatory events induce production of proinflammatory cytokines in the hippocampus, followed by impairments in spatial memory and learning [39].

In the present study, it was shown that long-term administration of glucosylceramide ameliorated the memory decline and anti-inflammatory activity in aged mice. The memory enhancing effect of glucosylceramide was confirmed in the Y-maze test, novel objective test, and Morris water maze task in different behavioral observation. In the Morris water maze test, glucosylceramide increased the swimming time within the target quadrant. The decrease in escape latency from day to day in the first trial represents long-term memory or reference memory [19]. The time in the quadrant with the platform reflects changes in spatial memory [40]. These results suggest that glucosylceramide improves the long-term memory in amnesic mouse models induced by ageing.

Mice fed with the control diet displayed decreasing spatial memory performances with age. This was confirmed to increase markers of inflammation such as iNOS and COX-2 as well as mRNA levels in the hippocampus and inhibit hippocampal-dependent working memory. However, administration of glucosylceramide decreased the age-associated expression of proinflammatory mediators such as iNOS and COX-2 in the aged mice brain and performance in the hippocampal-dependent working memory task was restored. Thus, age-related conditions that are exacerbated by inflammation, including cognitive aging and neurodegenerative diseases, may be inhibited by dietary intake of glucosylceramide. Also pretreatment of glucosylceramide suppressed the LPS-induced proinflammatory responses; iNOS, COX-2, and cytokines in mice.

Ceramide has been known as a sphingolipid with potent proinflammatory and proapoptotic properties [41–43] and its active metabolite, ceramide 1-phosphate, stimulates macrophage proliferation through activation of the PI3-kinase, JNK, and ERK pathways [44]. However, studies using primary cultures of neurons demonstrated that ceramide has multiple functions, depending on the cell type and the developmental stage. In immature hippocampal neurons, ceramide plays bipotential roles in cell survival and dendrite outgrowth in a dose-dependent manner [45]. It was also found that ceramide prevents cell death of motoneurons cultures through inhibition of oxidative signals [46]. These results suggest that the cellular level of ceramide is critical for regulation of neuronal survival and differentiation. In our previous studies, phytoceramide inhibited the glutamate-induced neurotoxicity in cultured neuronal cells, while phytosphingosine did not show the neuroprotective effect with the same dose of phytoceramide in the cultured neuronal cells [13]. There are some structural differences between phytoceramide and phytosphingosine. Phytosphingosine is structurally similar to sphingosine, except that phytosphingosine has a hydroxyl group at C-4 of the sphingoid long-chain base instead of the *trans*-double bond between C-4 and C-5. There is an interesting report that phytoceramide activates peroxisome proliferator-activated receptors (PPARs), whereas ceramide and dihydroceramide do not change the PPAR activity [47].

The structural feature of glucosylceramide is represented as attached glucose on terminal hydroxyl group while the normal ceramide consists of sphingoid base linked to a fatty acid with an amide bond. In this point the glucosylceramide would be hydrolyzed by glycosidases in vivo to generate ceramide. Even though general ceramide is formed as potential component in biosynthesis of many complex sphingolipids, the biological activities of ceramide showed relatively variable activity depending on the cell types. Glucosylceramide had better solubility than that of ceramide and showed better bioavailability. Interestingly, phytoceramide and glucosylceramide (after hydrolysis) have three –OH groups but ceramide has two hydroxyl groups. 

Collectively, glucosylceramide plays an important role in anti-inflammatory and memory enhancement, and it could be a potential new therapeutic agent for the treatment of dementia.
